# The short-term effects of wearing swimming goggles on corneal biomechanics

**DOI:** 10.1007/s10792-022-02268-8

**Published:** 2022-04-04

**Authors:** Raimundo Jiménez, Rubén Molina, Jesús Vera, Beatriz Redondo

**Affiliations:** CLARO (Clinical and Laboratory Applications of Research in Optometry) Research Group, Department of Optics, University of Granada, Campus de la Fuentenueva 2, 18071 Granada, Spain

**Keywords:** Corvis ST, Corneal deformation, Intraocular pressure, Central corneal thickness

## Abstract

**Purpose:**

This study aimed to assess the impact of wearing swimming goggles (SG) on corneal biomechanics.

**Methods:**

Corneal deformation response, central corneal thickness (CCT), intraocular pressure (IOP) and biomechanically corrected intraocular pressure (bIOP) were measured with the Corvis system (Oculus Optikgeräte GmbH, Wetzlar, Germany) in thirty-one healthy young adults while wearing a drilled SG. All measurements were obtained before, at 30 s, 2 min, 3.5 min and 5 min of wearing SG, just after SG removal and after 2 min of SG removal.

**Results:**

The corneal biomechanics is sensitive to SG wear, observing lower corneal deformability during SG use. Specifically, wearing SG caused an increase in the time and length of the first applanation and radius curvature at the highest concavity, as well as a decrease and in the velocity of the first applanation and time and deformation amplitude of the second applanation (*p* < 0.001 in all cases). After SG removal, corneal biomechanical parameters showed a rebound-effect, obtaining a higher corneal deformability in comparison with baseline reading (*p*-corrected < 0.05 in all cases). Additionally, IOP and bIOP significantly increased while wearing SG (*p* < 0.001 in both cases), whereas CCT remained stable (*p* = 0.850).

**Conclusions:**

Wearing SG modifies the biomechanical properties of the cornea, with reduced corneal deformability during SG wear. The outcomes of this study should be taken into consideration when making clinical decisions in subjects at high risk of developing corneal ectasias or glaucoma, as well as in the post-surgical management of these ocular conditions.

## Introduction

Biomechanics is often defined as mechanics applied to biology [1], and due to the viscoelastic characteristics of the cornea, it is possible to determine its biomechanical behaviour after applying a given force [2]. The clinical application of the corneal biomechanics has gained attention in the last years [3–5]. The assessment of corneal biomechanics has special relevance for the diagnosis, prognosis and treatment planning of different ocular conditions such as corneal ectatic disorders or glaucoma [4, 5], as well as for improving the safety and effectiveness of different ocular treatments or refractive surgical techniques [6–11].

There is a relationship between alterations in corneal biomechanics and different factors and ocular parameters such as age [12, 13], diabetes [14], caffeine intake [15], hormonal changes [16], level of hydration and fasting [17], exposure to ultraviolet radiation [18], intraocular pressure (IOP) [13, 19], refractive error [20], axial length [13], corneal central thickness and corneal curvature [13], ocular surgery [9, 10, 21] or orthokeratology [22, 23] among others.

In addition, activities that are known to increase IOP, such as eye rubbing and psychological stress, have also been associated with changes in the biomechanical properties of the cornea [24, 25]. In this regard, the use of swimming goggles (SG) has been shown to acutely increase IOP; this effect is attributed to the tension transmitted by the goggle headband and eye cups which compresses the orbital tissues and vasculature and consequently, modifying the eye dimensions [26, 27]. In a previous study, we assessed the impact of wearing SG on the anterior segment morphology, using the Pentacam system, and an acute corneal thinning, iridocorneal angle reduction and intraocular pressure elevation were observed [28]. However, to date, the impact of wearing SG on corneal biomechanical properties remains unknown.

In view of these limitations, the present study aimed to determine the short-term effect of wearing SG on the corneal biomechanics, as measured with corneal visualisation Scheimpflug technology, which has a good repeatability and reproducibility to analyse the corneal biomechanics in vivo and potential applicability in clinical settings [29–31]. Based on the previously mentioned evidence showing acute changes in IOP and eye anterior morphology with the use of SG [26, 28], and the well-proven association between IOP and corneal biomechanics [32], we hypothesised that SG wearing would alter the biomechanical properties of the cornea. Assessment of corneal biomechanics responses to habits or dailies activities such as SG wear may provide guidelines for the eye care community in order to minimise the risk for the development and progression of corneal ectasias or glaucoma, especially in high-risk individuals. A better understanding of the corneal biomechanical properties would also help to design strategies for the management of these ocular diseases at pre- and post-operative stages.

## Material and methods

### Participants and ethical approval

For sample size calculation, we performed an a priori power analysis, using the GPower 3.1 software [33], based on an expected low effect size (Cohen’s *d* = 0.20), and considering a power of 0.80 and alpha of 0.05. This calculation determined that 26 participants were required to achieve this desired level of accuracy. At this point, we recruited 31 healthy young adults (19 women; mean age ± standard deviation, 21.5 ± 1.9 years) to increase the statistical power and to account for possible drop out. Participants had a mean spherical equivalent of − 1.62 ± 1.28 D, ranging from 0 D to − 5.75 D. The study was carried out at the CLARO (Clinical and Laboratory Applications of Research in Optometry) laboratory located at the Faculty of Sciences of the University of Granada (Spain) from September 2019 to December 2019. The following inclusion criteria were considered: (1) no systemic or ocular disease, and not taking any medication; (2) no history of previous ocular surgery, trauma or orthokeratology; (3) baseline intraocular pressure value ≤ 21 mmHg [34]; and (4) to refrain for wearing contact lenses and consumption of alcohol/caffeine-based drinks for at least 8 h before attending to the experimental session. Informed consent was obtained from all participants, and the experimental protocol was approved by the University of Granada Institutional Review Board (IRB approval: 438/ CEIH/2017).

### Experimental design

**Fig. 1:**
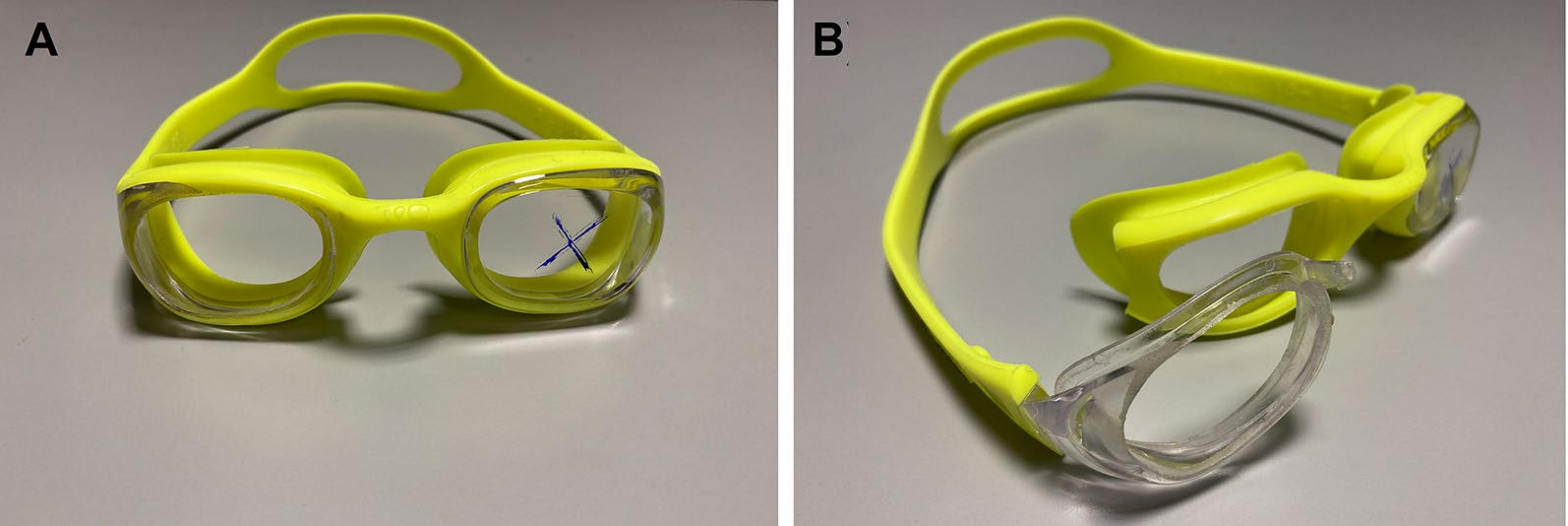
Photographs of the SG used in the study, showing that the right eye piece was partially drilled (panel **a**), although the structure of the cup was preserved (panel **b**)

All participants wore the same model of SG (Nabaiji, Decathlon Group Inc., Villeneuve d’Ascq, France). The SG consisted of two separated rigid plastic eye cups with a rubber cushioning seal surrounding the lip of each cup and a non-adjustable elastic strap. Vertical and horizontal goggles widths of the cup were 45 mm and 33 mm, respectively, from the internal rubber seals of each eyepiece. Based on the published studies of Paula et al. [35] and Jiménez et al. [28], we used a SG with part of the plastic right eye piece drilled, which allowed us to measure IOP and corneal biomechanics parameters while wearing the SG. Previous studies have evidenced that the impact of SG wear on the ocular physiology reversed immediately after the removal of the SG [28, 35], and thus, we considered appropriate to use a modified SG, since it was the only alternative to assess the eye changes during SG wear. The structure of the cup of the right eye piece was preserved to achieve a similar level of pressure to real-life conditions (see Fig. 1 for a photograph of the SG).

A repeated measures design was followed to evaluate the short-term effects of SG wearing on the biomechanical properties of the cornea. Also, the impact of SG wearing on IOP and CCT was assessed. The point of measurement (baseline, 30 s, 2 min, 3.5 min, 5 min, immediately after SG removal [recovery 1, *R*1] and 2 min after removal [recovery 2, *R*2]) was considered as the only within-participants factor. Each participant followed an identical protocol, and all measurements were taken by the same optometrist.

### Instruments and measurements

The biomechanical properties of the cornea were assessed by Corvis ST (Oculus Optikgeräte GmbH, Wetzlar, Germany) at seven points of measurement, before (baseline), during (30 s, 2 min, 3.5 min and 5 min) and after SG wearing (*R*1 and *R*2). This instrument used a non-contact tonometer based on air puff indentation. At the same time, a high-speed Scheimpflug camera took over 4000 frames per second, which allowed direct real-time visualisation of the corneal deformation response during an entire cycle after each air pulse. As a consequence, from the disturbed state by each air puff, the cornea firstly flattens (inward applanation), reaching the highest concavity, and again undergoes another applanation state (outward applanation) before becoming fully restored to its normal state. The CorVis ST provides a number of parameters, and for this study, we considered the time of the first and second applanations (A1T and A2T, respectively), the length of the first and second applanations (A1L and A2L, respectively), the amplitude of the first and second applanations (A1D and A2D, respectively), the velocity of the first and second applanations (A1V and A2V, respectively), the deformation amplitude at the highest concavity (HCDA), the time to highest concavity (HCT), the highest concavity curvature (HCR) and the peak distance (PD) (see Jiménez et al. [15] for a description of these measurements).

Additionally, measurements of the corneal central thickness (CCT), standard intraocular pressure (IOP, based on the inward applanation) and biomechanically corrected IOP value (bIOP) were obtained in each point of measurement. The bIOP is referred to as the IOP reading free from the effects of corneal parameters [36].

### Procedure

Upon arrival at the laboratory, participants read and signed the consent form, and completed a questionnaire with demographic information. Then, the baseline measurement with the CorVis ST was performed. Just after this, the participant wore the SG and the CorVis ST measurements were taken at 30 s, 2 min, 3.5 min and 5 min of SG wearing. Immediately after the last measurement, the SG was removed and a new Corvis ST measurement was taken (*R*1). Finally, after two minutes of SG removal, a new measurement was taken (*R*2). All measurements were taken from a randomly selected eye for each subject, and Corvis ST examinations with the quality score “OK” were always obtained.

### Statistical analysis

Normal distribution and homogeneity of variances were checked by the Shapiro–Wilk and Levene’s tests, respectively (*p* > 0.05). Then, separate repeated measures one-way analyses of variance (ANOVAs) with Holm–Bonferroni adjusted post hoc comparisons were carried out to assess the effect of SG use on corneal biomechanics (A1T, A1V, A1D, A1L, A2T, A2V, A2D, A2L, HCDA, HCT, HCR, PD), IOP, bIOP and CCT. For each dependent variable, the point of measurement (baseline, 30 s, 2 min, 3.5 min and 5 min; *R*1 and *R*2) was considered as the only within-participants factor. The partial eta-squared (*η*^2^) and Cohen’s d were reported to describe the magnitude of the differences for *F* and *T*-tests, respectively. The level of statistical significance was set at 0.05. The JASP statistics package (version 0.13.1.0) was used for statistical analyses.

## Results

Descriptive values (mean ± standard deviation) for all the measurements taken are depicted in Table 1.

**Table 1:** Average ± standard deviation values of intraocular pressure, central corneal thickness and corneal biomechanical parameters at the different points of measurement

	Baseline	Wearing swimming goggles				Recovery	
		30 s	2 min	3.5 min	5 min	Just after removal	2 min after removal
IOP (mmHg)	15.21 ± 2.19	17.18 ± 4.06	17.21 ± 4.43	17.18 ± 4.19	17.37 ± 3.27	13.95 ± 2.29	14.13 ± 1,97
bIOP (mmHg)	15.38 ± 1.86	17.25 ± 3.66	17.25 ± 3.73	17.16 ± 3.61	17.36 ± 3.05	14.25 ± 1.71	14.31 ± 1.60
CCT (µm)	540.92 ± 32.08	539.35 ± 32.73	540.51 ± 31.01	541.02 ± 30.41	540.41 ± 33.19	540.43 ± 32.86	542.06 ± 34.40
A1T (ms)	7.36 ± 0.31	7.66 ± 0.57	7.64 ± 0.60	7.64 ± 0.56	7.67 ± 0.47	7.21 ± 0.29	7.22 ± 0.26
A1V (m/s)	0.14 ± 0.02	0.12 ± 0.02	0.12 ± 0.02	0.12 ± 0.02	0.12 ± 0.02	0.15 ± 0.02	0.15 ± 0.02
A1L (mm)	2.22 ± 0.16	2.37 ± 0.27	2.44 ± 0.39	2.43 ± 0.33	2.50 ± 0.40	2.24 ± 0.20	2.25 ± 0.12
A1D (mm)	0.12 ± 0.01	0.13 ± 0.01	0.13 ± 0.01	0.13 ± 0.02	0.13 ± 0.01	0.12 ± 0.01	0.12 ± 0.01
A2T (ms)	22.00 ± 0.39	21.42 ± 0.82	21.63 ± 0.49	21.55 ± 0.57	21.56 ± 0.46	22.20 ± 0.81	22.12 ± 0.77
A2V (m/s)	− 0.29 ± 0.03	− 0.28 ± 0.08	− 0.30 ± 0.05	− 0.30 ± 0.04	− 0.30 ± 0.05	− 0.31 ± 0.06	− 0.31 ± 0.04
A2L (mm)	2.68 ± 0.59	2.93 ± 0.38	2.84 ± 0.52	3.12 ± 0.73	3.13 ± 0.76	2.65 ± 0.53	2.72 ± 0.52
A2D (mm)	0.37 ± 0.06	0.33 ± 0.06	0.33 ± 0.05	0.34 ± 0.05	0.34 ± 0.05	0.36 ± 0.05	0.36 ± 0.05
HCDA (mm)	1.08 ± 0.15	1.01 ± 0.11	1.05 ± 0.15	1.06 ± 0.12	1.05 ± 0.13	1.17 ± 0.17	1.16 ± 0.15
HCT (ms)	16.68 ± 1.30	16.54 ± 0.75	16.77 ± 1.05	16.71 ± 0.89	16.70 ± 0.98	16.80 ± 0.47	16.90 ± 0.39
HCR (mm)	7.31 ± 0.67	8.08 ± 1.09	7.61 ± 0.89	7.65 ± 1.17	7.77 ± 1.42	6.99 ± 1.14	7.14 ± 0.63
PD (mm)	5.16 ± 0.26	5.03 ± 0.32	5.09 ± 0.32	5.09 ± 0.26	5.10 ± 0.25	5.39 ± 0.46	5.31 ± 0.35

IOP: non-corrected intraocular pressure, bIOP: biomechanically corrected intraocular pressure, CCT: central corneal thickness, A1T: time of the first applanation, A1V: velocity of the first applanation, A1L: length of the first applanation, A1D: amplitude of the flattened area in the inward applanation, A2T: time of the first applanation, A2V: velocity of the first applanation, A2L: length of the first applanation, A2D: amplitude of the flattened area in the outward applanation, HCDA: maximum deformation amplitude of the cornea at the highest concavity, HCT: time for reaching the highest concavity, HCR: central curvature radius at the highest concavity, PD: distance between the two apexes at the highest concavity

### Intraocular pressure

**Fig. 2:**
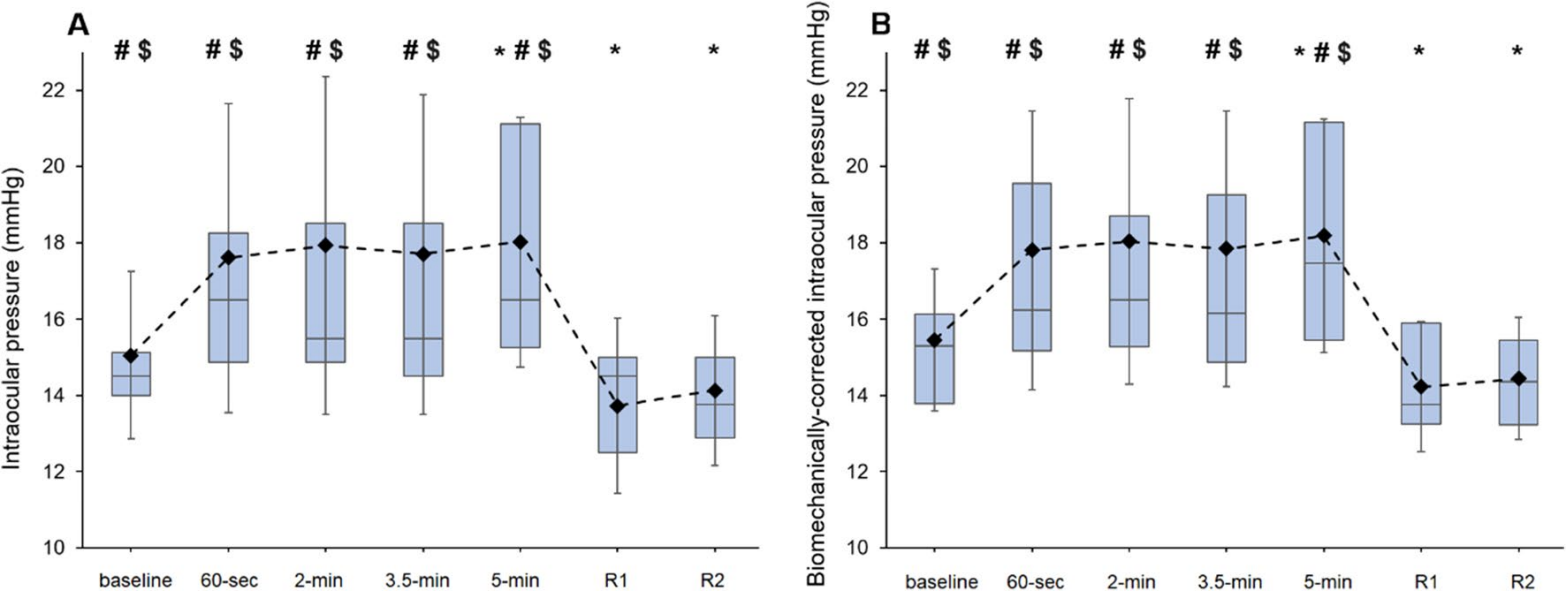
Effects of SG wear on non-corrected intraocular pressure (panel **a**) and biomechanically corrected IOP (panel **b**) at the different points of measurement. The box plots represent 75th and 25th centiles. Horizontal lines and diamonds symbols into the box represent median and mean values, respectively. The whiskers show the standard deviation. *, # and $ denote statistically significant differences (corrected *p* values < 0.05) when compared with the baseline, just after removal (*R*1) and after 2 min of recovery (*R*2), respectively. All values are calculated across participants (*n* = 31)

We found statistically significant differences for IOP and bIOP while wearing the SG (*F*_6_,_180_ = 11.14, *p* < 0.001, *η*^2^ = 0.27 and *F*_6_,_180_ = 11.35, *p* < 0.001, *η*^2^ = 0.28, respectively). For IOP, post hoc analyses demonstrated greater IOP values at 5 min in comparison with the baseline measurement (*p *corrected = 0.025, *d* = 0.60). Higher IOP readings were also obtained at baseline, 30 s, 2 min, 3.5 min and 5 min in comparison with the measurement obtained immediately after SG removal (*p*-corrected = 0.01, *d* = 0.68; *p*-corrected = 0.008, *d* = 0.71; *p*-corrected = 0.005, *d* = 0.75; *p*-corrected = 0.01, *d* = 0.68; and *p*-corrected < 0.001, *d* = 0.88, respectively), as well as after 2 min of recovery (*p*-corrected = 0.007, *d* = 0.72; *p*-corrected = 0.008, *d* = 0.70; *p*-corrected = 0.006, *d* = 0.73; *p*-corrected = 0.013, *d* = 0.65; and *p*-corrected < 0.001, *d* = 0.88, respectively). Similarly, post hoc analyses for bIOP evidenced a heightened IOP response after 5 min of SG wear in comparison with the baseline measurement (*p*-corrected = 0.027, *d* = 0.60). In addition, there were higher IOP values at baseline, 30 s, 2 min, 3.5 min and 5 min when compared to the *R*1 (*p*-corrected = 0.009, *d* = 0.68; *p*-corrected = 0.007, *d* = 0.70; *p*-corrected = 0.004, *d* = 0.75; *p*-corrected = 0.01, *d* = 0.67; and *p*-corrected < 0.001, *d* = 0.86, respectively) and *R*2 (*p*-corrected = 0.002, *d* = 0.80; *p*-corrected = 0.006, *d* = 0.72; *p*-corrected = 0.004, *d* = 0.75; *p*-corrected = 0.011, *d* = 0.66; and *p*-corrected < 0.001, *d* = 0.90) measurements (Fig. 2).

### Central corneal thickness

The use of SG was far from having a statistically significant effect on CCT (*F*_6_,_180_ = 0.442, *p* = 0.850).

### Corneal biomechanics parameters

In regard to the first applanation, there was a main effect of the point of measurement for A1T, A1V, A1L and A1D (*F*_6_,_180_ = 11.39, *p* < 0.001, *η*^2^ = 0.28; *F*_6_,_180_ = 26.42, *p* < 0.001, *η*^2^ = 0.47; *F*_6_,_180_ = 6.846, *p* < 0.001, *η*^2^ = 0.19; and *F*_6_,_180_ = 2.728, *p* = 0.015, *η*^2^ = 0.08, respectively).

**Fig. 3:**
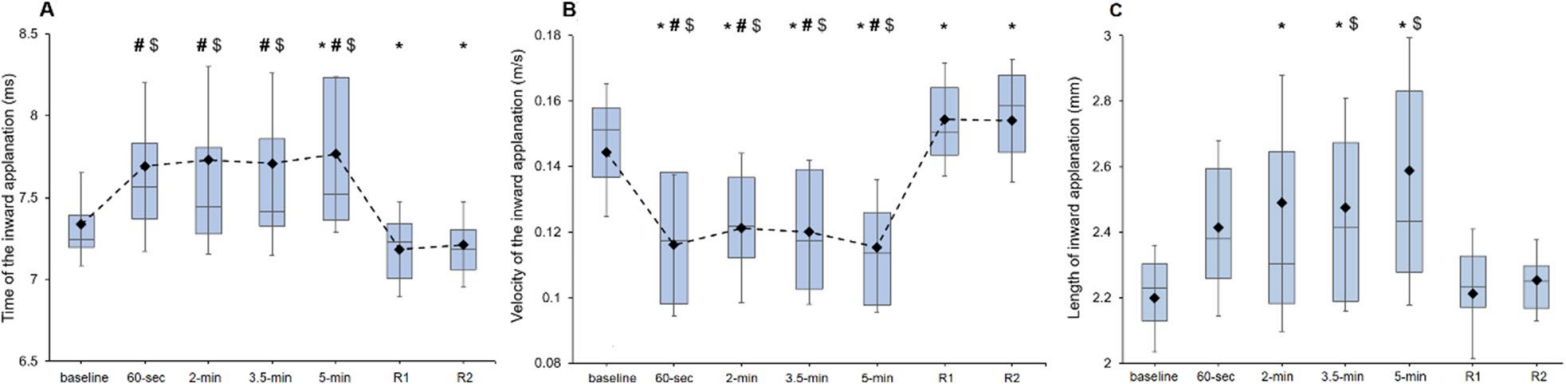
Effects of SG wear on A1T (panel **a**), A1V (panel **b**) and A1L (panel **c**) at the different points of measurement. The box plots represent 75th and 25th centiles. Horizontal lines and diamonds symbols into the box represent median and mean values, respectively. The whiskers show the standard deviation. *, # and $ denote statistically significant differences (corrected *p* values < 0.05) when compared with the baseline, just after removal (*R*1) and after 2 min of recovery (*R*2), respectively. All values are calculated across participants (*n* = 31)

Post hoc comparisons only showed a significant increase in A1T at 5 min of SG use in comparison with the baseline measurement (*p*-corrected = 0.020, *d* = 0.62). The A1T value obtained immediately after SG removal (*R*1) was lower than the measurements taken at baseline, 30 s, 2 min, 3.5 min and 5 min (*p*-corrected = 0.023, *d* = 0.61; *p*-corrected = 0.007, *d* = 0.71; *p*-corrected = 0.004, *d* = 0.75; *p*-corrected = 0.011, *d* = 0.68; and *p*-corrected < 0.001, *d* = 0.87, respectively). In a similar manner, A1T was lower after 2 min of SG removal in comparison with the baseline (*p*-corrected = 0.023, *d* = 0.61), 30 s (*p*-corrected = 0.007, *d* = 0.71), 2 min (*p*-corrected = 0.004, *d* = 0.75), 3.5 min (*p*-corrected = 0.011, *d* = 0.68) and 5 min (corrected *p* value < 0.001, *d* = 0.87) measurements (Fig. 3, panel A).

For A1V, post hoc analyses revealed a decrease while SG wear in comparison with the baseline value (*p*-corrected = 0.001, *d* = 0.81 at 30 s; *p*-corrected = 0.007, *d* = 0.67 at 2 min; *p*-corrected = 0.005, *d* = 0.70 at 3.5 min; and *p*-corrected < 0.001, *d* = 0.90 at 5 min). The *R*1 and *R*2 measurements were also higher than those taken at baseline (*p*-corrected = 0.006, *d* = 0.68; and *p*-corrected = 0.005, *d* = 0.71, respectively), 30 s (*p*-corrected < 0.001, *d* = 1.21; and *p*-corrected < 0.001, *d* = 1.15, respectively), 2 min (*p*-corrected < 0.001, *d* = 1.13; and *p*-corrected < 0.001, *d* = 1.02, respectively), 3.5 min (*p*-corrected < 0.001, *d* = 1.12; and *p*-corrected < 0.001, *d* = 1.14, respectively) and 5 min (*p*-corrected < 0.001, *d* = 1.40; and *p*-corrected < 0.001, *d* = 1.29, respectively) of SG wear (Fig. 3, panel B). For its part, post hoc tests for A1L displayed an increase at 2 min, 3.5 min and 5 min of SG wear in comparison with the baseline measurement (*p*-corrected = 0.025, *d* = 0.64; *p*-corrected < 0.017, *d* = 0.676; and *p*-corrected = 0.029, *d* = 0.62, respectively). After 2 min of SG removal, A1L values were lower than the measurement taken at 3.5 min and 5 min of SG wear (*p*-corrected = 0.046, *d* = 0.59; and *p*-corrected = 0.011, *d* = 0.70, respectively) (Fig. 3, panel C). Lastly, post hoc for A1D showed no significant changes in any post hoc comparison (*p*-corrected > 0.05 in all cases).

**Fig. 4:**
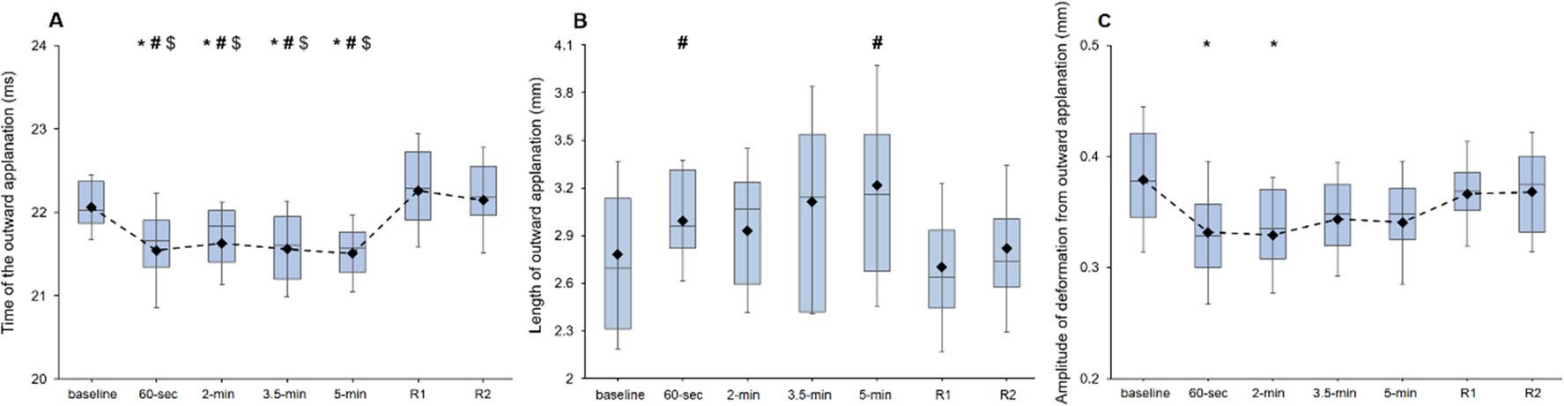
Effects of SG wear on A2T (panel **a**), A2L (panel **b**) and A2D (panel **c**) at the different points of measurement. The box plots represent 75th and 25th centiles. Horizontal lines and diamonds symbols into the box represent median and mean values, respectively. The whiskers show the standard deviation. *, # and $ denote statistically significant differences (corrected *p* values < 0.05) when compared with the baseline, just after removal (*R*1) and after 2 min of recovery (*R*2), respectively. All values are calculated across participants (*n* = 31)

Regarding parameters related to the second applanation, there was also a main effect of the point of measurement for A2T, A2V, A2L and A2D with the SG (*F*_6_,_180_ = 11.60, *p* < 0.001, *η*^2^ = 0.28; *F*_6_,_180_ = 2.16, *p* = 0.049, *η*^2^ = 0.07; *F*_6_,_180_ = 5.60, *p* < 0.001, *η*^2^ = 0.16; *F*_6_,_180_ = 6.47, *p* < 0.001, *η*^2^ = 0.18, respectively). Post hoc comparisons showed a decrease in A2T during SG wearing at 30 s, 2 min, 3.5 min and 5 min in comparison with the baseline reading (*p*-corrected = 0.014, *d* = 0.66; *p*-corrected = 0.002, *d* = 0.81; *p*-corrected = 0.014, *d* = 0.67; and *p*-corrected = 0.001, *d* = 0.85, respectively). The recovery values (*R*1 and *R*2) were significantly higher than the measurements taken at 30 s (*p*-corrected < 0.001, *d* = 0.93; and *p*-corrected < 0.001, *d* = 0.85, respectively), 2 min (*p*-corrected = 0.014, *d* = 0.65; and *p*-corrected = 0.030, *d* = 0.58, respectively), 3.5 min (*p*-corrected = 0.014, *d* = 0.66; and *p*-corrected = 0.021, *d* = 0.61, respectively) and 5 min (*p*-corrected = 0.008, *d* = 0.70; and *p*-corrected = 0.014, *d* = 0.64, respectively) of SG wear (Fig. 4, panel A). Post hoc analyses for A2V showed no changes between any comparison (*p*-corrected > 0.05 in all cases). For A2L, there were greater values at 30 s and 5 min of SG wear in comparison with the measurement taken immediately after SG removal (*p*-corrected = 0.028, *d* = 0.64; and *p*-corrected = 0.048, *d* = 0.60, respectively) (Fig. 4, panel B). The analysis of A2D revealed lower values at 30 s and 2 min of SG wear in comparison with the baseline measurement (*p*-corrected = 0.015, *d* = 0.67; and *p*-corrected = 0.011, *d* = 0.70, respectively) (Fig. 4, panel C).

**Fig. 5:**
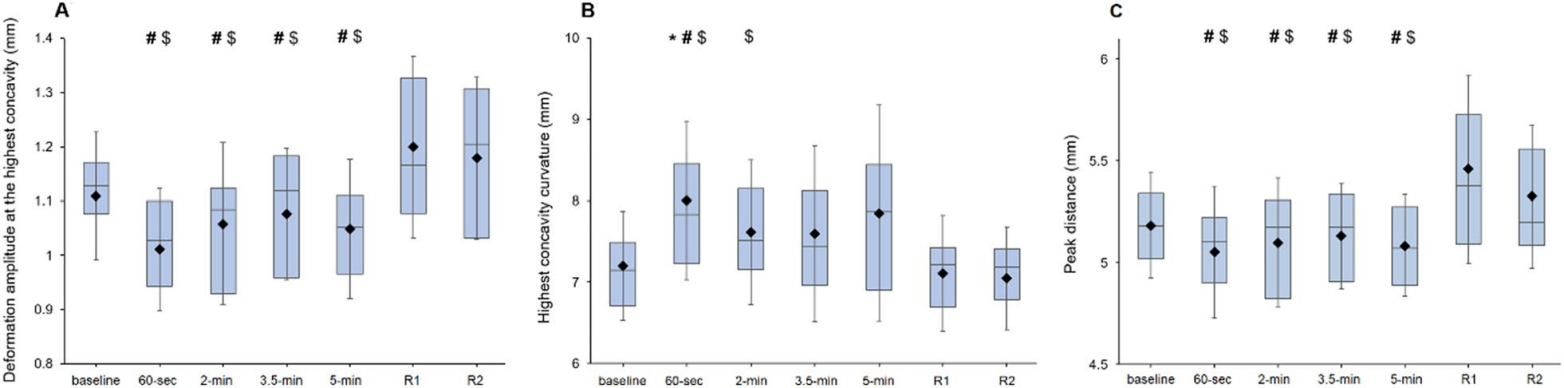
Effects of SG wear on HCDA (panel **a**), HCR (panel **b**) and PD (panel **c**) at the different points of measurement. The box plots represent 75th and 25th centiles. Horizontal lines and diamonds symbols into the box represent median and mean values, respectively. The whiskers show the standard deviation. *, # and $ denote statistically significant differences (corrected *p* values < 0.05) when compared with the baseline, just after removal (*R*1) and after 2 min of recovery (*R*2), respectively. All values are calculated across participants (*n* = 31)

HCDA showed statistical significance for the point of measurement (*F*_6_,_180_ = 11.00, *p* < 0.001, *η*^2^ = 0.27). Post hoc comparisons evidenced a higher HCDA when the SG was removed (*R*1) in comparison with the baseline value and measurements taken while wearing the SG (baseline, 30 s, 2 min, 3.5 min and 5 min; *p*-corrected = 0.024, *d* = 0.61; *p*-corrected < 0.001, *d* = 0.86; *p*-corrected = 0.005, *d* = 0.74; *p*-corrected = 0.008, *d* = 0.70; and *p*-corrected = 0.001, *d* = 0.83, respectively), as well as after 2 min of SG removal (baseline, 30 s, 2 min, 3.5 min and 5 min; *p*-corrected < 0.023, *d* = 0.62; *p*-corrected < 0.001, *d* = 0.90; *p*-corrected = 0.008, *d* = 0.70; *p*-corrected = 0.014, *d* = 0.65; and *p*-corrected = 0.002, *d* = 0.80, respectively) (Fig. 5, panel A).

There was also a main effect of the point of measurement for HCR (*F*_6_,_180_ = 6.778, *p* < 0.001, *η*^2^ = 0.18), with post hoc analyses showing statistically significant differences for the comparison baseline versus 30 s of SG wear (*p*-corrected = 0.017, *d* = 0.67), 30 s of SG wear versus *R*1 (*p*-corrected = 0.019, *d* = 0.65), 30 s of SG wear versus *R*2 (*p*-corrected < 0.001, *d* = 0.86) and 2 min of SG wear versus *R*2 (*p*-corrected < 0.022, *d* = 0.64) (Fig. 5 panel B).

For its part, PD revealed statistically significant differences for the point of measurement (*F*_6_,_180_ = 9.40, *p* < 0.001, *η*^2^ = 0.24). Post hoc comparisons evidenced a higher PD immediately after SG removal in relation to all the previously taken measurements (baseline, 30 s, 2 min, 3.5 min and 5 min; *p*-corrected = 0.016, *d* = 0.65; *p*-corrected = 0.011, *d* = 0.69; *p*-corrected = 0.008, *d* = 0.71; *p*-corrected = 0.016, *d* = 0.66; and *p*-corrected = 0.005, *d* = 0.75, respectively), as well as after 2 min of SG removal comparison with the baseline (*p*-corrected < 0.002, *d* = 0.82), 30 s (*p*-corrected = 0.019, *d* = 0.62), 2 min (*p*-corrected = 0.018, *d* = 0.64), 3.5 min (*p*-corrected = 0.018, *d* = 0.64) and 5 min (*p*-corrected = 0.002, *d* = 0.80) measurements (Fig. 5, panel C). Lastly, HCT was far from showing statistically significant differences for the point of measurement (*F*_6_,_180_ = 0.558, *p* = 0.764).

## Discussion

Our study assessed the short-term effects of SG wear on the biomechanical properties of the cornea in a healthy young population. Complementarily, the IOP behaviour and CCT were also examined. There have been a number of studies have reported changes in the eye physiology with the use of SG [26, 28], however, to the best of our knowledge, this is the first study to evaluate the corneal biomechanical behaviour during and after SG wear. We found significant alterations in corneal biomechanics and IOP, suggesting that the use of SG causes a reduction in corneal deformability. Also, greater values of both non-corrected and biomechanically corrected IOP were observed while wearing the SG, but no changes were found for CCT.

There is scientific evidence that IOP suffers alterations with the use of SG, namely IOP increases during and after SG wear regardless of the physical structure of SG and duration of their use [26, 28, 37, 38]. Our findings agree with these results, since we found an average IOP rise of approximately 2 mmHg during SG wear and rapidly returning to baseline values after SG removal. Due to the fact that IOP measurements may be influenced by the biomechanical properties of the cornea, bIOP values based on the first applanation were also considered [36]. Similarly, a significant bIOP increase was observed while SG wear. Therefore, as indicated by Morgan et al. [26], it is plausible that the mechanical pressure exerted by the SG on the orbital tissue compress the ocular globe and consequently could lead to a significant increase in IOP.

Wearing SG has also demonstrated to induce acute changes in the ocular biometrics. In this regard, previous studies have reported an increase in axial length and a reduction in CCT and iridocorneal angle [27]. In a recent study from this research group, using the Pentacam system, we found an increase in IOP and a reduction in CCT while wearing a drilled SG [28]. However, this finding did not fully agree with our results, since we found an elevation in both IOP and bIOP measurements without changes in CCT, corroborating that bIOP from the Corvis ST is independent of CCT [39]. Somewhat surprisingly, although we used the same SG of our previous study, we did not observe a reduction in CCT, which could be explained by the different instruments used in both studies. The Pentacam is based on a 360-degree rotating Scheimpflug camera which acquires images that contain measurement points on the front and back corneal surfaces to determine a true elevation map, and the Corvis ST, also based on ultra-high-speed Scheimpflug technology, acquires the measurements during the deformation process but only in the horizontal meridian. Although a high repeatability and agreement in CCT measurements have been reported for healthy eyes [40–42], it is plausible to expect that the ocular deformation induced by the SG may differ in different corneal meridians. The mechanical pressure exerted by the SG on the ocular globe may be stronger in the vertical than the horizontal direction, but this hypothesis needs to be tested in future studies.

Regarding corneal biomechanics, our results showed smaller values of A1V, A2T, A2D and higher of A1T, HCR during SG wear, which could indicate that SG wearing causes a higher corneal stiffness. Accordingly, a less deformable cornea reaches the first applanation faster, shows a smaller concavity and reaches the second applanation slower [43]. Based on these results, SG wear seems to enhance corneal stiffness, but these changes rapidly recover after SG removal. Nevertheless, as reported above, the mechanical pressure exerted by the goggles on the orbital tissue and adjacent scleral tissue may be different depending corneal direction considered, leading to unequal changes in viscoelasticity in the corneal stroma in different meridians [44–47]. However, due to technical characteristics of the Corvis ST, this possibility cannot be discerned with the current results, which required the assessment of the corneal biomechanics along different meridians.

Remarkably, A1T, A1V, A1L, A2D, HCDA, HCR and PD exhibited a rebound effect when the SG was removed, with this change lasting for at least 2 min. We consider that it would be of interest to test how much time is needed to stabilise the corneal biomechanics after SG wear. (e.g. eye rubbing, lid massages, sleep face down or SG wearing), which would allow preventing adverse consequences for ocular health. Additionally, it is important that eye care specialists are mindful of these results to minimise confounding factors in clinical decision-making. The current findings may be of special relevance in the short- and long-term management of clinical populations with alterations in the corneal biomechanics (e.g. corneal ectatic disorders or glaucoma). Future studies should examine the risks associated with the use of different SG designs in these individuals, aiming to provide recommendations about the most pertinent type of SG, if any, for the minimisation of ocular side effects.

Several limitations should be acknowledged in this study in order to make a correct interpretation of the current outcomes. First, the generalisability of our findings is potentially limited since a greater pressure on ocular tissues may occur with an intact SG (without drilling the plastic eye cup) and with the influence of external water pressure upon goggles. Also, all measurements were taken in a resting state, and therefore, the effects of wearing SG on IOP and corneal biomechanics could differ with physical activity. Second, we used a specific type of SG and other goggle designs could cause different effects on the biomechanical properties of the cornea to those observed in this investigation. Third, there are claims that corneal stiffness is age-dependent [48, 49], and further studies with a larger sample size are needed to determine the effect of SG wear on corneal biomechanics in different age groups. Fourth, some ocular measurements (e.g. scleral rigidity, anterior chamber angle, anterior chamber depth, etc.) have demonstrated to differ between hyperopic and myopic eyes [50–52]. In the current study, the experimental sample was formed by emmetropes and myopes, and it did not allow us to compare the corneal biomechanics changes caused by wearing swimming goggles between myopic and hyperopic eyes. Lastly, healthy subjects were included in this study, and thus, our results should be cautiously interpreted in clinical populations (e.g. individuals with corneal ectasias or glaucoma patients), who have demonstrated to have an altered corneal deformation response [53, 54].

In conclusion, our data revealed changes in most corneal biomechanical parameters while wearing SG, showing a heightened corneal stiffness with the use of SG. These effects may be due to modifications in the viscoelastic properties of the cornea to a given force. The assessment of corneal deformation responses in other ocular meridians would help to better characterise the corneal biomechanics changes induced by SG. The current results may be of interest for the management of ocular conditions that are known to be tightly linked to the biomechanical properties of the cornea (e.g. corneal ectasias and glaucoma).
